# ^1^H, ^13^C and ^15^N resonance assignments of the Cdc42-binding domain of TOCA1

**DOI:** 10.1007/s12104-016-9677-8

**Published:** 2016-03-18

**Authors:** Joanna R. Watson, Daniel Nietlispach, Darerca Owen, Helen R. Mott

**Affiliations:** Department of Biochemistry, University of Cambridge, 80 Tennis Court Road, Cambridge, CB2 1GA UK

**Keywords:** TOCA1, HR1, Cdc42, GTPase, Cytoskeleton, Endocytosis, Signalling

## Abstract

TOCA1 is a downstream effector protein of the small GTPase, Cdc42. It is a multi-domain protein that includes a membrane binding F-BAR domain, a homology region 1 (HR1) domain, which binds selectively to active Cdc42 and an SH3 domain. TOCA1 is involved in the regulation of actin dynamics in processes such as endocytosis, filopodia formation, neurite elongation, cell motility and invasion. Structural insight into the interaction between TOCA1 and Cdc42 will contribute to our understanding of the role of TOCA1 in actin dynamics. The ^1^H, ^15^N and ^13^C NMR backbone and sidechain resonance assignment of the HR1 domain (12 kDa) presented here provides the foundation for structural studies of the domain and its interactions.

## Biological context

Transducer of Cdc42-dependent Actin assembly protein 1 (TOCA1) is a downstream effector of the small GTPase, Cdc42 (Ho et al. 2004). Cdc42 is a member of the Rho family of small G proteins, which are in turn, members of the Ras superfamily. The Rho family are particularly associated with regulation of actin dynamics, and bind to a number of effector proteins to achieve their functions. TOCA1 binding to Cdc42 is involved in the activation of another Cdc42 effector, N-WASP (the ubiquitously expressed homologue of the Wiskott–Aldrich Syndrome Protein, WASP) in *Xenopus tropicalis* extracts. The importance of TOCA1 in actin dynamics has been confirmed in a range of systems (Bu et al. 2009, 2010; Lee et al. 2010; Gallop et al. 2013). Furthermore, TOCA1 has been implicated in membrane invagination and endocytosis (Tsujita et al. 2006; Fricke et al. 2009; Giuliani et al. 2009; Bu et al. 2010; Bai and Grant 2015), filopodia formation (Bu et al. 2009), neurite elongation (Kakimoto et al. 2006) and cell motility and invasion (Hu et al. 2011; Chander et al. 2012).

TOCA1 is a multidomain protein of the Pombe Cdc15 Homology (PCH) family. It has an N-terminal membrane binding domain (F-BAR), a central Cdc42-binding domain (HR1) and a C-terminal SH3 domain. The F-BAR domain binds to and deforms PI(4,5)P_2_-containing membranes (Itoh et al. 2005; Henne et al. 2007; Bu et al. 2009). The HR1 domain binds to Cdc42 (Ho et al. 2004), an interaction suggested to promote F-BAR-dependent membrane deformation (Bu et al. 2009). The SH3 domain has many known binding partners, including N-WASP (Ho et al. 2004) and the endocytosis protein, Dynamin (Itoh et al. 2005). It has been suggested that the TOCA1 HR1 domain may bind Cdc42 simultaneously with the N-WASP G protein-binding domain (GBD) (Ho et al. 2004; Bu et al. 2010). However, there is currently no direct evidence for such a complex. Structural insight into the TOCA1-Cdc42 interaction is of upmost importance in answering the question of whether these two effector proteins bind Cdc42 simultaneously.

In addition to its importance in understanding the pathway of actin dynamics, this resonance assignment will also be generally interesting with regards to G protein signalling. For example, HR1 domains show differential selectivity for specific G proteins. The HR1 domains from TOCA1 and from another TOCA family member, CIP4 bind to Cdc42 (Aspenström 1997; Ho et al. 2004) whereas the HR1 domains from the protein kinase C-related kinase (PRK) family show differential specificity for Rac1, RhoA, RhoB and RhoC (Modha et al. 2008; Hutchinson et al. 2013). Further structural analysis of G protein-HR1 domain interactions is therefore of significant interest.

The ^1^H, ^15^N and ^13^C NMR resonance assignment of the TOCA1 HR1 domain, presented here, will provide the foundation for structure determination and for studying its interaction with Cdc42. It will also be useful in determining whether TOCA1 and N-WASP bind Cdc42 simultaneously. The resonance assignment is, therefore, of importance for better understanding the pathways of Cdc42-dependent actin dynamics and in general for gaining understanding of G protein-effector interactions.

## Materials and experiments

### Protein cloning, expression, labelling and purification

The TOCA1 HR1 domain (residues 330-426 based on sequence alignments) was cloned from full-length *Xenopus tropicalis* TOCA1 cDNA, into the *Bam*HI and *Eco*RI sites of pGEX-6P-1 (Addgene). The construct was expressed as a GST-fusion in *E. coli* BL21 cells (Invitrogen). Stationary cultures were diluted 1 in 10 and grown at 37 °C until an A_600_ ~ 0.8 was reached, then induced with 0.1 mM isopropyl-β-d-thiogalactopyranoside for 20 h at 20 °C. To produce isotopically labelled protein for NMR studies, cells were grown in M9 media supplemented with ^15^NH_4_Cl and ^13^C-glucose (Sigma-Aldrich). The proteins were purified using glutathione-agarose beads (Sigma), and eluted from the beads by cleavage of the GST-tag by HRV 3C protease prior to gel filtration on a 16/60 S75 column (GE Healthcare).

### NMR spectroscopy

All NMR experiments were run at 25 °C with 0.9 mM ^13^C/^15^N-labelled HR1 domain in 20 mM sodium phosphate pH 7.5, 150 mM NaCl, 5 mM MgCl_2_, 5 mM DTT, 10% D_2_O). ^15^N-HSQC, ^15^N-separated NOESY (100 ms mixing time), ^15^N-separated TOCSY (48 ms mixing time), HNCA (Nietlispach et al. 2002), HNCO, HN(CO)CA, HNCACB and HN(CO)CACB (reviewed in Ferentz and Wagner 2000) were recorded on a Bruker DRX500. A ^13^C-separated HCCH-TOCSY (FLOPSY-16 mixing, 18.6 ms mixing time) and ^13^C-separated NOESY (100 ms mixing time) were recorded on an Avance AV600. NMR data were processed using *AZARA* (W. Boucher).

Standard methodology (Cavanagh et al. 2006) was used to carry out the backbone assignment with reference to 2D ^15^N-HSQC and ^13^C-HSQC experiments using ^15^N-separated NOESY and TOCSY experiments, 3D HNCA, HNCO, HN(CO)CA, HNCACB and HN(CO)CACB in *ANALYSIS* (Vranken et al. 2005). The 3D HCCH-TOCSY and ^13^C-separated NOESY were used in addition to these experiments to assign the sidechain resonances.

## Resonance assignment and data deposition

**Fig. 1:**
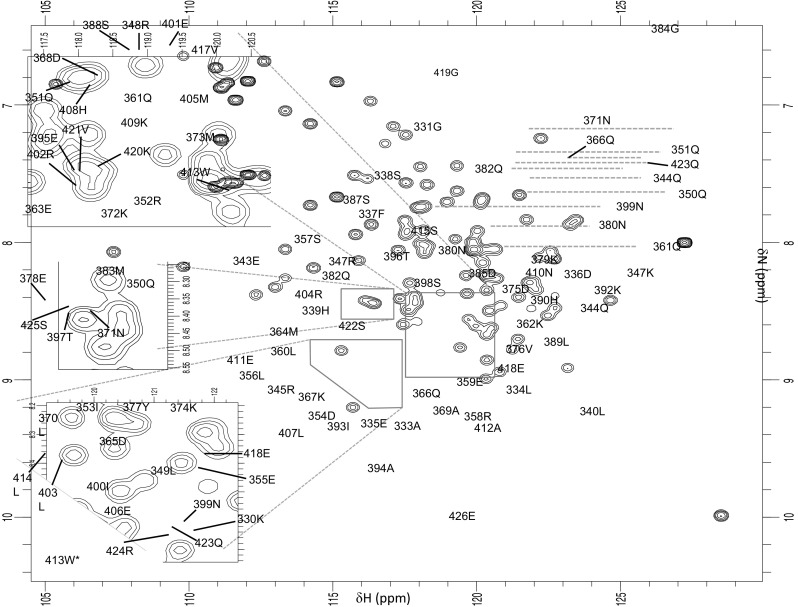
A ^15^N-HSQC recorded on ^13^C, ^15^N-labelled TOCA1 HR1 domain in 20 mM sodium phosphate pH 7.5, 150 mM NaCl, 5 mM DTT, 5 mM MgCl_2_, recorded at 500 MHz at 25 °C

The TOCA1 HR1 domain construct gave well-dispersed spectra under the conditions described in "Materials and experiments" section (Fig. 1). The construct comprised residues 330-426 of TOCA1 with five additional residues at the N-terminus encoded by the expression vector (GPLGS). Assignment of 85 % of all ^15^N, ^1^H and ^13^C atoms in the construct was achieved. Excluding the five additional N-terminal residues, which are not of interest, 97.9 % of the backbone resonances have been assigned. This includes 100 % of the amide groups. The carbonyl carbons of Ser425, Glu426 and of the residues preceding the six Prolines, for which no HNCO transfer is possible, account for all of the unassigned backbone resonances. All of the observable sidechain resonances have been assigned, equating to 94.3 % of all sidechain NH and CH groups. The resonances of Lysine Nζ, Arginine Nη and Histidine Nδ and Nε were not observed for any of these residues. These resonances are rarely observable in NMR spectra. The NHε groups of Arginines 348, 358, 402, 404 and 424 were also not observed. These account for the remaining 5.7 % of unassigned resonances. The backbone ^1^H, ^13^C, and ^15^N chemical shifts were validated using TALOS-N (Shen and Bax 2013) before being deposited at the BioMagResBank (accession number 25945).

**Fig. 2:**
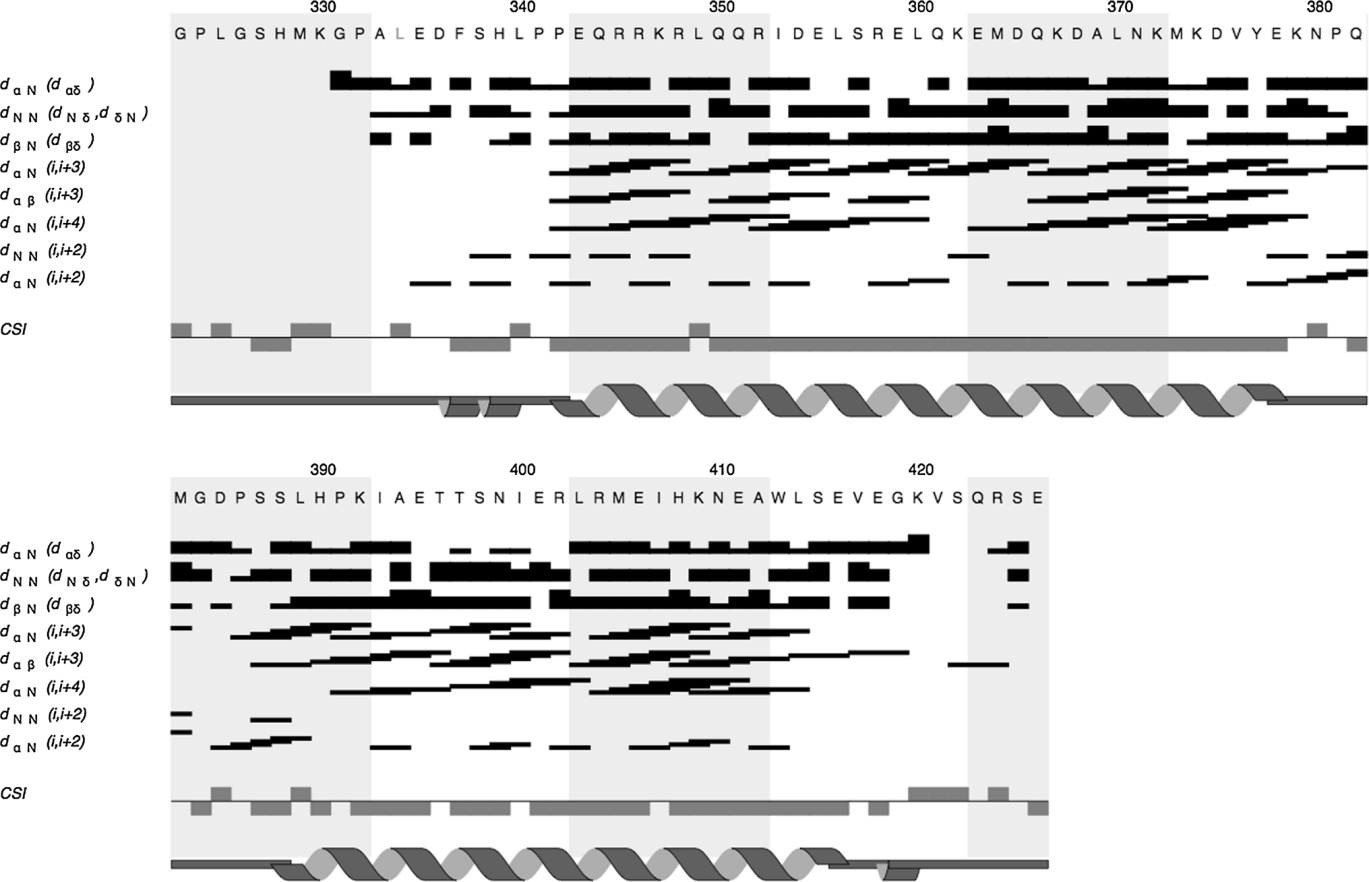
Secondary structure summary of the TOCA1 HR1 domain. *Black bars* indicate NOEs for each of the atoms listed. The *height* of the *bar* represents the strength of the NOE. The atoms listed depict the following NOEs: d_αN_ (d_αδ_) = C^α^H of residue i and the NH of residue i + 1, or C^δ^H of i + 1 in the case of Prolines; d_NN_(d_Nδ_,d_δN_) = NH of residue i and the NH of residue i + 1, or C^δ^H in the case of Prolines; d_βN_ (dβδ) = C^β^H of residue i and either the NH or C^δ^H (Prolines) of residue i + 1. For the last four rows, d_xy_ (i,i + z) is used, where x and y denote the atoms in residues i and i + z respectively. The Chemical Shift Index value (CSI) is calculated from all of the backbone atom chemical shifts. A value of +1 indicates a β-strand and a value of −1 indicates an α-helix. The secondary structure was predicted from the chemical shifts using TALOS-N (Shen and Bax 2013) and is depicted as a cartoon below the CSI. This figure was generated in CCPN *ANALYSIS* (Vranken et al. 2005)

The difference in backbone chemical shifts (Cα, Cβ, and CαH) from the random coil positions were used to generate the Chemical Shift Index (CSI), which can be used to predict the secondary structure. The short-range NOEs and the CSI for each residue are shown in Fig. 2. The predicted secondary structure, predicted based on chemical shifts using TALOS-N (Shen and Bax 2013) is also shown. The data indicate that the TOCA1 HR1 domain comprises two α-helices separated by a region of no defined secondary structure. This is consistent with previously studied HR1 domains, which are known to adopt an anti-parallel coiled-coil fold (Maesaki et al. 1999; Modha et al. 2008; Kobashigawa et al. 2009). The completeness of this HR1 domain assignment will allow for structure determination using distance restraints derived from further NMR experiments as well as detailed investigation of its interaction with Cdc42. This information will be used to test the models of the role of the HR1 domain of TOCA1 in its regulation of actin dynamics.
